# Supplementary material for: Development of Pleiotropic TrkB and 5-HT4 Receptor Ligands as Neuroprotective Agents
Source: Molecules. 2024 Jan 19;29(2):515. doi: 10.3390/molecules29020515 (PMC10819171; doi:10.3390/molecules29020515)

Supplementary Materials

Development of Pleiotropic TrkB and 5-HT_4 Receptor Ligands as Neuroprotective Agents

Mirjana Antonijevic [1,†], Despoina Charou [2,3,†], Audrey Davis [1], Thomas Curel [4], Maria Valcarcel [5], Isbaal Ramos [5], Patricia Villacé [5], Sylvie Claeysen [4], Patrick Dallemagne [1], Achille Gravanis [2,3], Ioannis Charalampopoulos [2,3] and Christophe Rochais [1,*]

[1] Normandie University, Unicaen, Centre d'Etudes et de Recherche sur le Médicament de Normandie (CERMN), 14000 Caen, France; mirjana.antonijevic@unicaen.fr (M.A.); audrey.davis@unicaen.fr (A.D.); patrick.dallemagne@unicaen.fr (P.D.)
[2] Department of Pharmacology, Medical School, University of Crete, 70013, Heraklion, Greece; dcharou@gmail.com (D.C.); gravanis@med.uoc.gr (A.G.); charalampn@uoc.gr (I.C.)
[3] Institute of Molecular Biology & Biotechnology, Foundation of Research & Technology-Hellas, 70013 Heraklion, Greece
[4] IGF, Univ Montpellier, CNRS, INSERM, 34000 Montpellier, France; thomas.curel@igf.cnrs.fr (T.C.); sylvie.claeysen@igf.cnrs.fr (S.C.)
[5] Innoprot S.L, Derio, 48160 Bizkaia, Spain; mvalcarcel@innoprot.com (M.V.); iramos@zabala.es (I.R.); pvillace@innoprot.com (P.V.)
* Correspondence: christophe.rochais@unicaen.fr; Tel.: +33-2-31-56-68-13
† These authors contributed equally to this work.

Contents

Analytical spectrum of compounds 7 – 19 2
Full images of Western blot of phosphorylation assay of NIH-3T3 TrkB cell line 15
Determination of cAMP production 19

Analytical spectrum of compounds 7 – 19

1,3-dimethyl-5-[(2-{4-[3-(4-amino-5-chloro-2-methoxyphenyl)-3-oxopropyl]piperidin-1-yl}ethyl)carbamoyl]benzene-1,3-dicarboxylate carbamate (7)

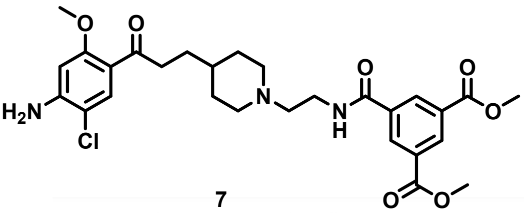

7

Elemental Composition Report Page

Single Mass Analysis
Tolerance = 5.0 PPM / DBE: min = -1.5, max = 50.0
Element prediction: Off
Number of isotope peaks used for i-FIT = 3

Monoisotopic Mass, Even Electron Ions
13720 formula(e) evaluated with 1 results within limits (up to 50 best isotopic matches for each mass)
Elements Used:
C: 28-28 H: 35-35 B: 0-1 N: 0-200 O: 0-200 Na: 0-1 Cl: 0-8
MAC042 187 (1.710) Cm (180:216)
1: TOF MS ES+

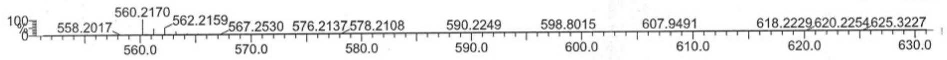

Minimum:				-1.5				
Maximum:		5.0	5.0	50.0				
Mass	Calc. Mass	mDa	PPM	DBE	i-FIT	Norm	Conf(%)	Formula
560.2170	560.2164	0.6	1.1	12.5	1604.2	n/a	n/a	C28 H35 N3 O7 Cl

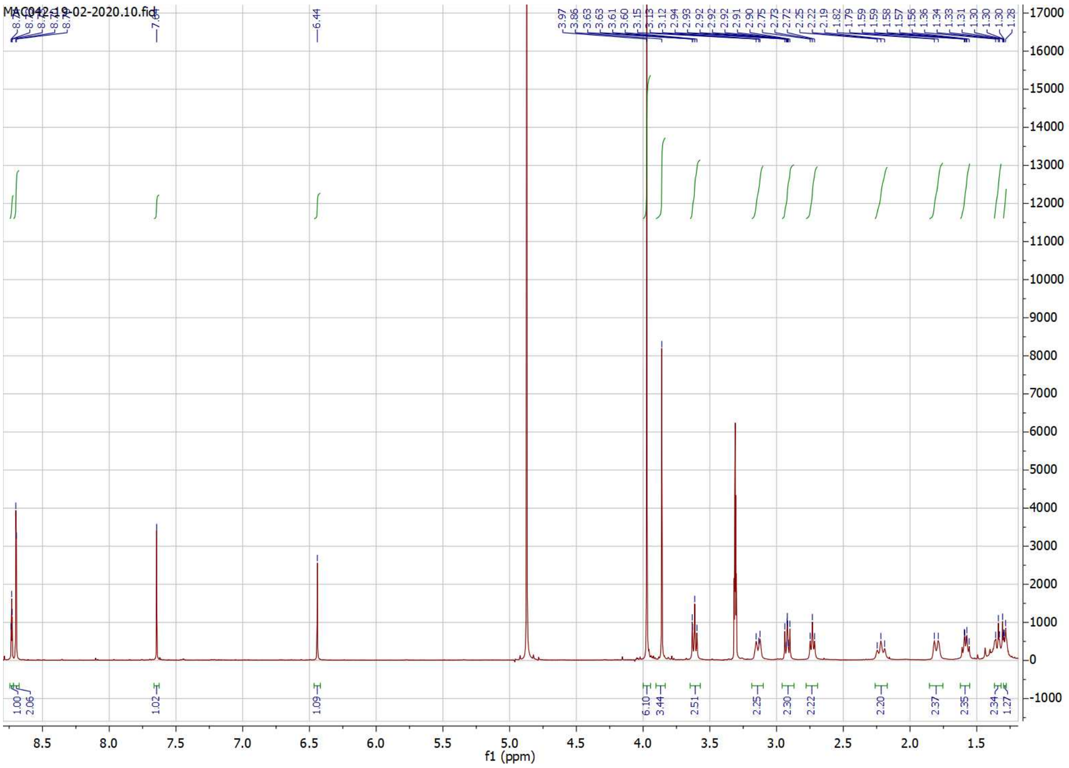

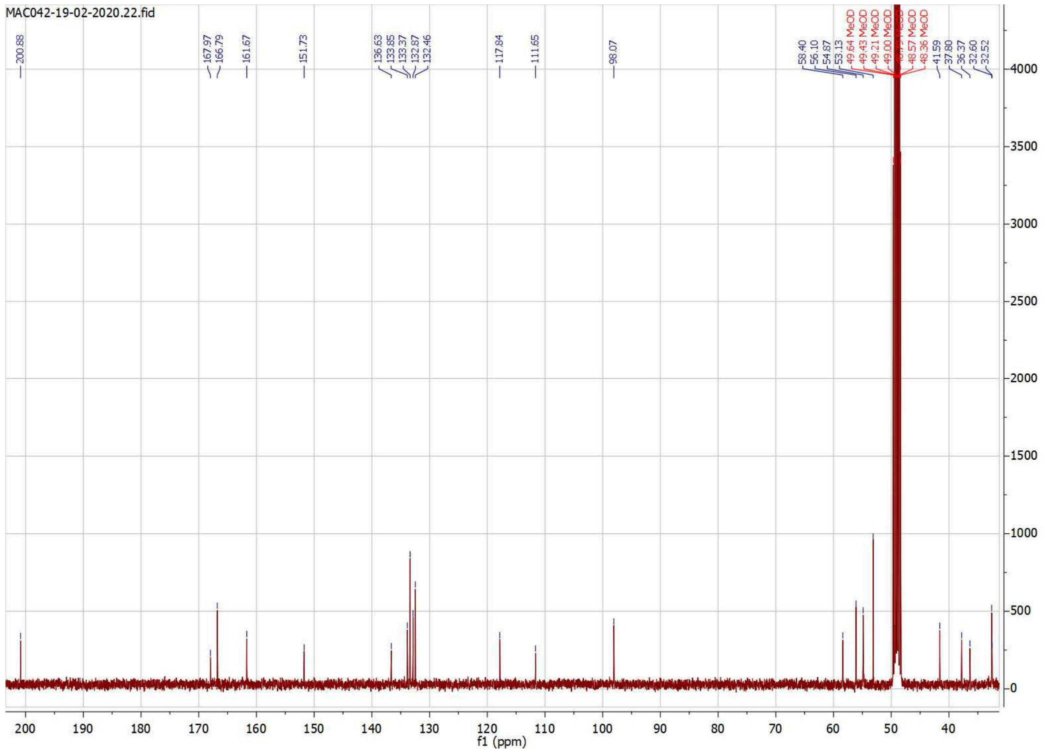

N^1^-(2-{4-[3-(4-amino-5-chloro-2-methoxyphenyl)-3-oxopropyl]piperidin-1-yl}ethyl)-N^3^,N^5^-bis(2-hydroxyethyl)benzene-1,3,5-tricarboxamide (8)

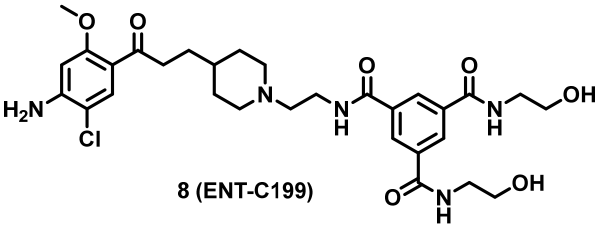

8 (ENT-C199)

Elemental Composition Report

Page 1

Single Mass Analysis

Tolerance = 5.0 PPM / DBE: min = -1.5, max = 50.0
Element prediction: Off
Number of isotope peaks used for i-FIT = 5

Monoisotopic Mass, Even Electron Ions
150 formula(e) evaluated with 1 results within limits (up to 50 best isotopic matches for each mass)
Elements Used:
C: 0-30 H: 0-1000 N: 0-6 O: 0-8 35Cl: 0-1

CERMN_MAC_043 145 (1.364) Cm (144:145-(135:139+203:210))
1: TOF MS ES+

1.79e+004

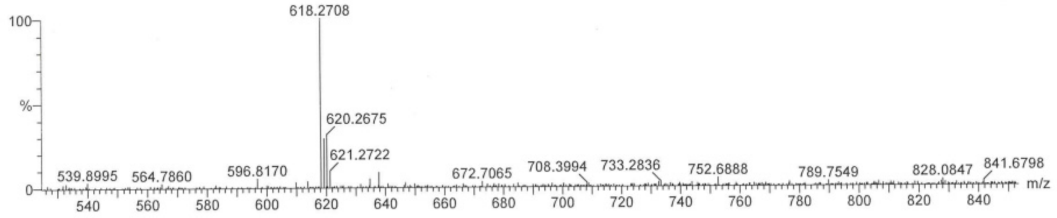

Minimum:				-1.5				
Maximum:		5.0	5.0	50.0				
Mass	Calc. Mass	mDa	PPM	DBE	i-FIT	Norm	Conf(%)	Formula
618.2708	618.2695	1.3	2.1	12.5	72.1	n/a	n/a	C30 H41 N5 O7 35Cl

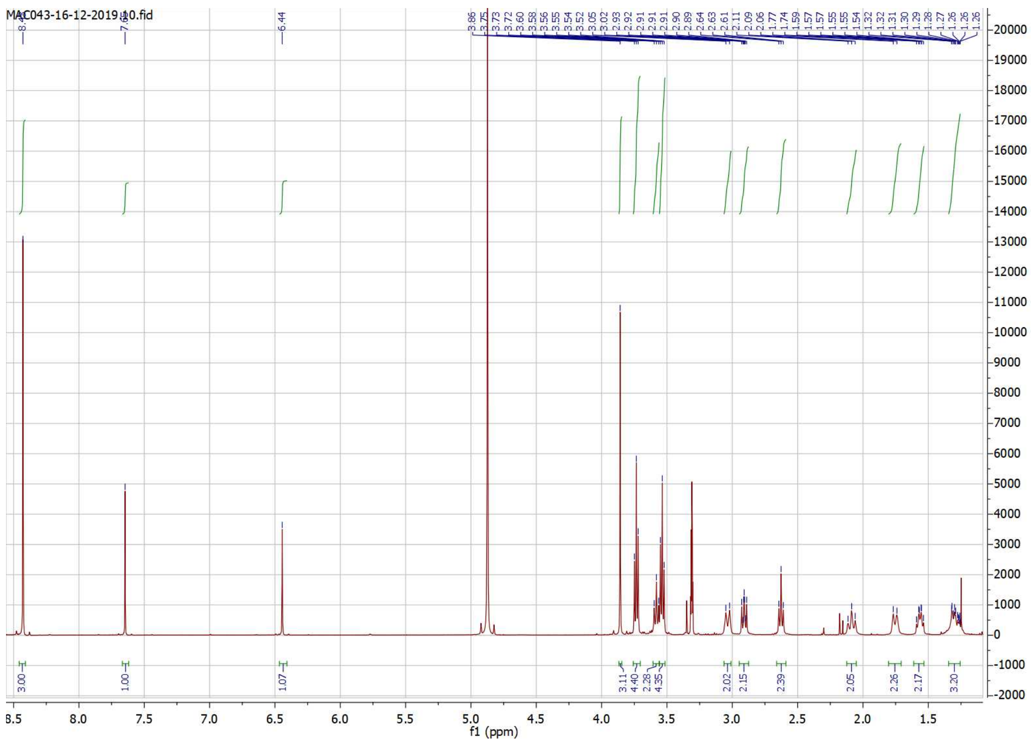
MAC043-16-12-2019.10.fid
f1 (ppm)

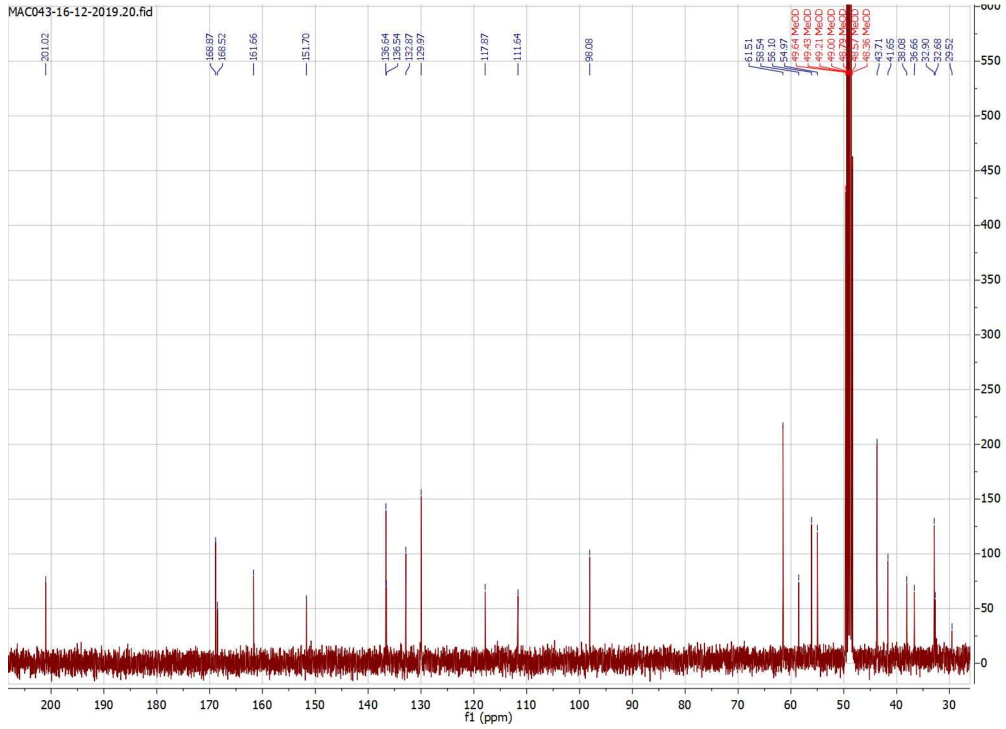
MAC043-16-12-2019.20.fid
f1 (ppm)

Tert-butyl-(3-(4-(3-(4-amino-5-chloro-2-methoxyphenyl)-3-oxopropyl)piperidin-1-yl)propyl)carbamate (11)

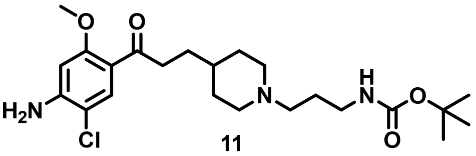

11

Elemental Composition Report **Page 1**

Single Mass Analysis
Tolerance = 5.0 PPM / DBE: min = -1.5, max = 50.0
Element prediction: Off
Number of isotope peaks used for i-FIT = 3

Monoisotopic Mass, Even Electron Ions
323 formula(e) evaluated with 1 results within limits (up to 50 closest results for each mass)
Elements Used:
C: 0-31 H: 0-100 N: 0-4 O: 0-6 Cl: 0-1
MAC076 181 (1.698) Cm (181:186)
1: TOF MS ES+

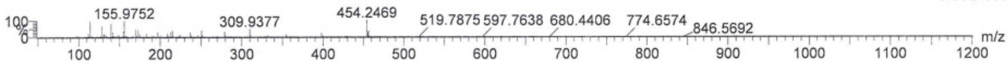

Minimum:				-1.5				
Maximum:		5.0	5.0	50.0				
Mass	Calc. Mass	mDa	PPM	DBE	i-FIT	Norm	Conf(%)	Formula
454.2469	454.2473	-0.4	-0.9	6.5	1716.8	n/a	n/a	C23 H37 N3 O4 Cl

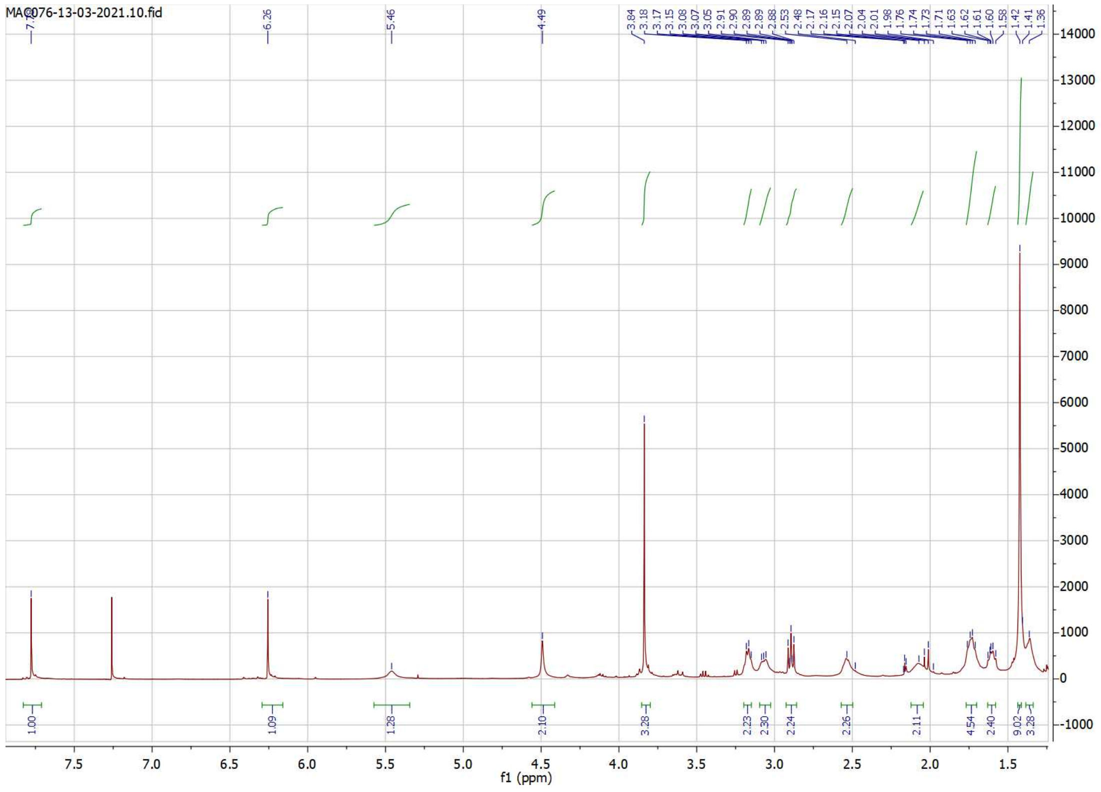

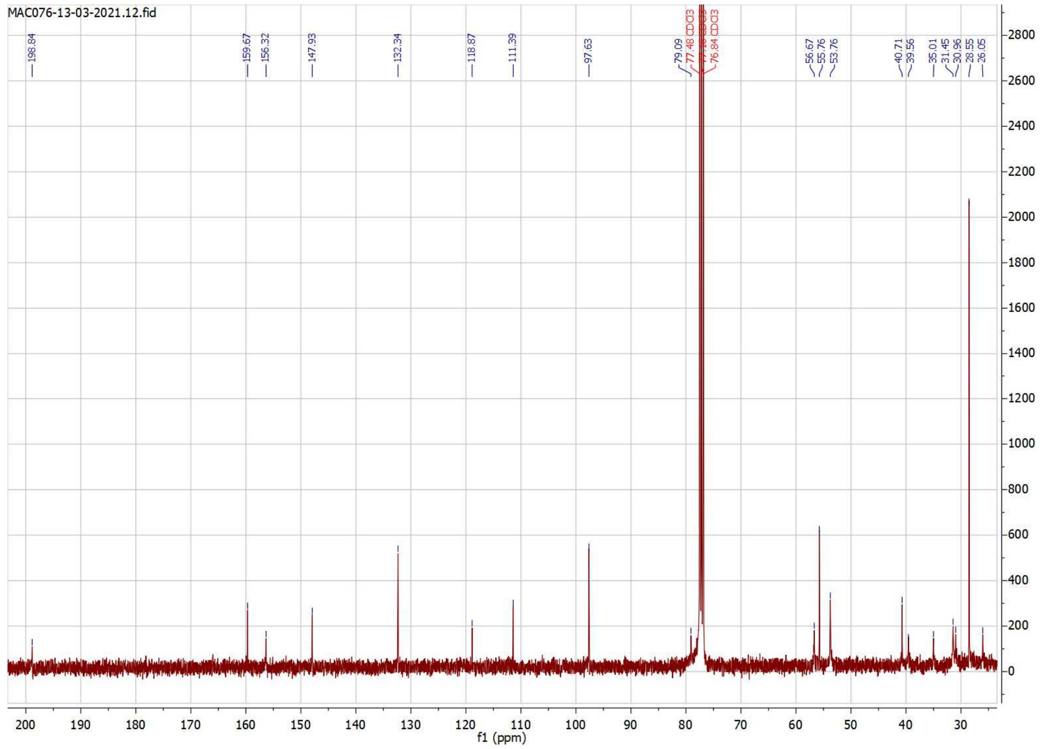

1,3-dimethyl-5-[(2-{4-[3-(4-amino-5-chloro-2-methoxyphenyl)-3-oxopropyl]piperidin-1-yl}propyl)carbamoyl]benzene-1,3-dicarboxylate (12)

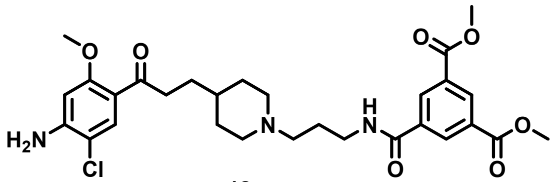

12

Elemental Composition Report **Page 1**

Single Mass Analysis
Tolerance = 5.0 PPM / DBE: min = -1.5, max = 50.0
Element prediction: Off
Number of isotope peaks used for i-FIT = 3

Monoisotopic Mass, Even Electron Ions
123 formula(e) evaluated with 1 results within limits (up to 50 closest results for each mass)
Elements Used:
C: 0-31 H: 0-100 N: 0-4 O: 0-7 Cl: 0-1
MAC078 230 (2.146) Cm (230:237)
1: TOF MS ES+

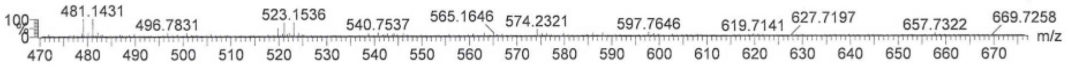

Minimum:				-1.5				
Maximum:		5.0	5.0	50.0				
Mass	Calc. Mass	mDa	PPM	DBE	i-FIT	Norm	Conf(%)	Formula
574.2321	574.2320	0.1	0.2	12.5	1562.9	n/a	n/a	C29 H37 N3 O7 Cl

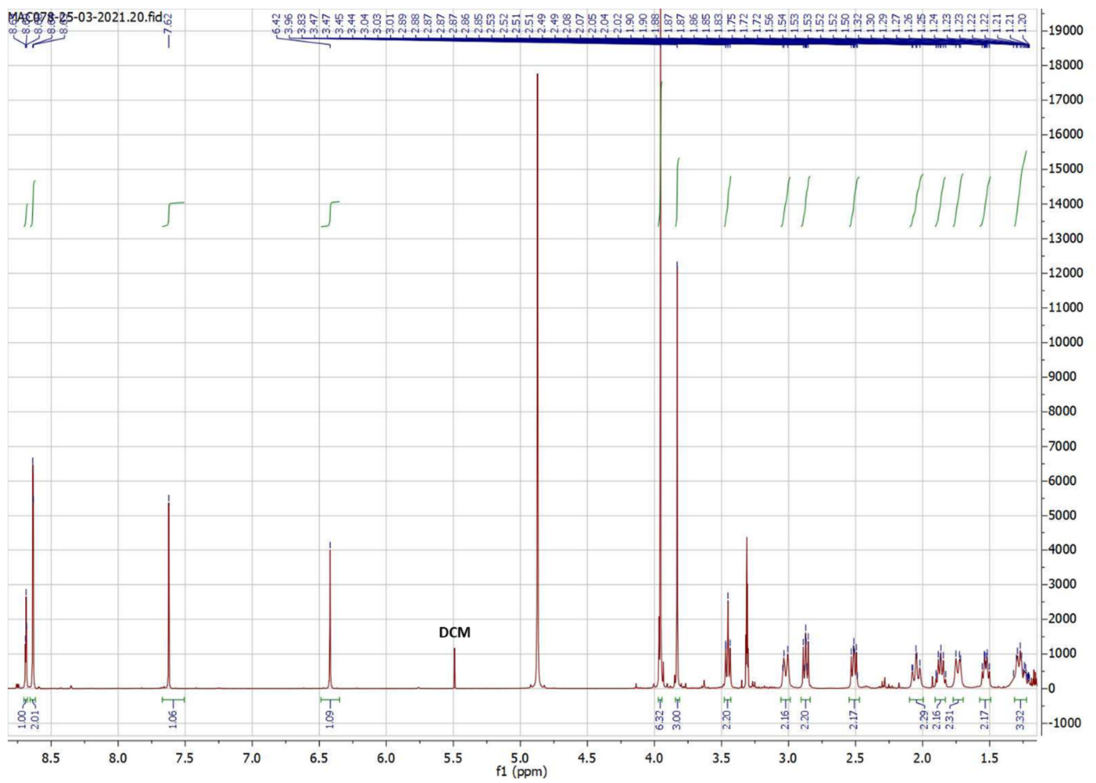
MAC078-25-03-2021.20.fid
DCM
f1 (ppm)

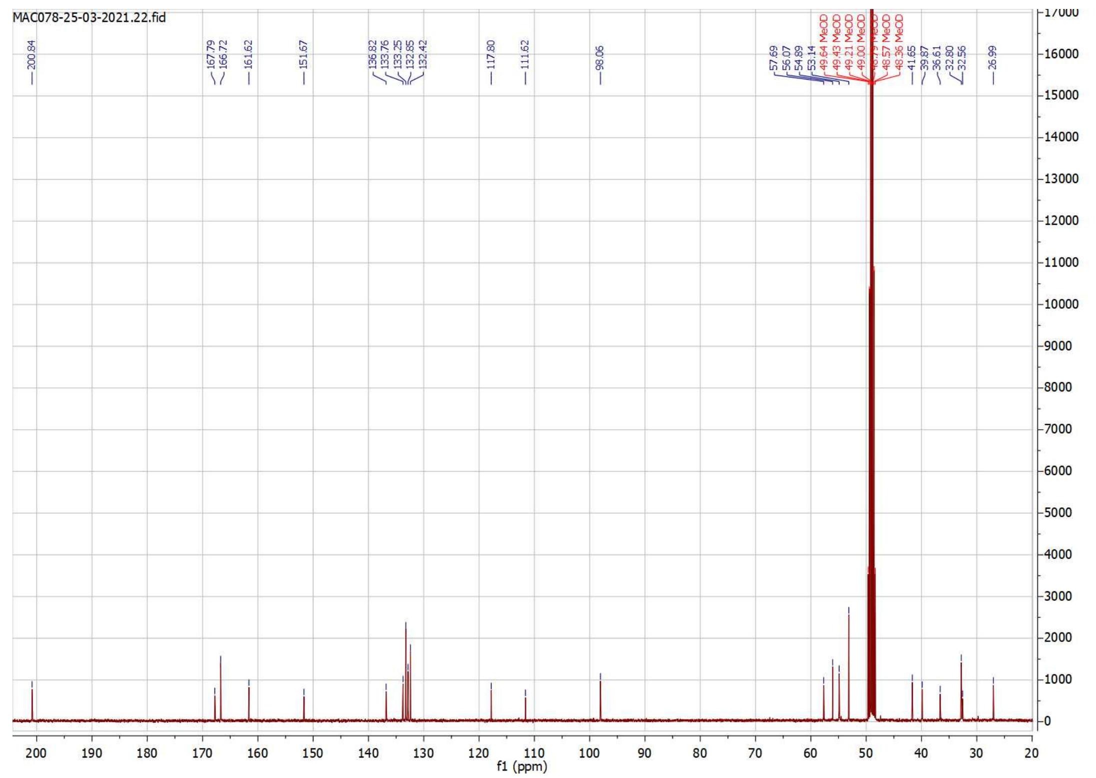
MAC078-25-03-2021.22.fid
200.84
167.79
166.72
161.62
151.67
136.82
133.76
133.25
132.85
132.42
117.80
111.62
98.06
57.69
56.07
54.89
53.14
41.65
39.87
36.61
32.80
32.56
26.99
f1 (ppm)

N[1]-(3-(4-(3-(4-amino-5-chloro-2-methoxyphenyl)-3-oxopropyl)piperidin-1-yl)propyl)-*N*[3],*N*[5]-bis(2-hydroxyethyl)benzene-1,3,5-tricarboxamide (13)*

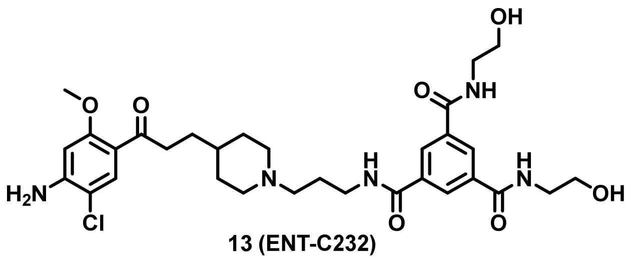

13 (ENT-C232)

Elemental Composition Report

Page 1

Single Mass Analysis

Tolerance = 5.0 PPM / DBE: min = -1.5, max = 50.0
Element prediction: Off
Number of isotope peaks used for i-FIT = 3

Monoisotopic Mass, Even Electron Ions
100 formula(e) evaluated with 1 results within limits (up to 50 closest results for each mass)
Elements Used:
C: 0-31 H: 0-100 N: 0-5 O: 0-7 Cl: 0-1
MAC079 148 (1.391) Cm (145:148)
1: TOF MS ES+

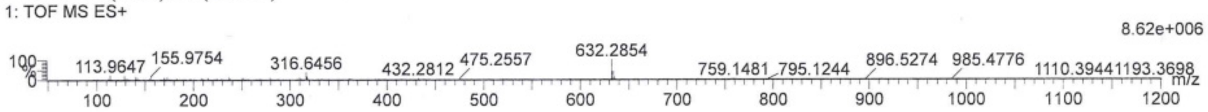

Minimum:				-1.5				
Maximum:		5.0	5.0	50.0				
Mass	Calc. Mass	mDa	PPM	DBE	i-FIT	Norm	Conf(%)	Formula
632.2854	632.2851	0.3	0.5	12.5	1507.7	n/a	n/a	C31 H43 N5 O7 Cl

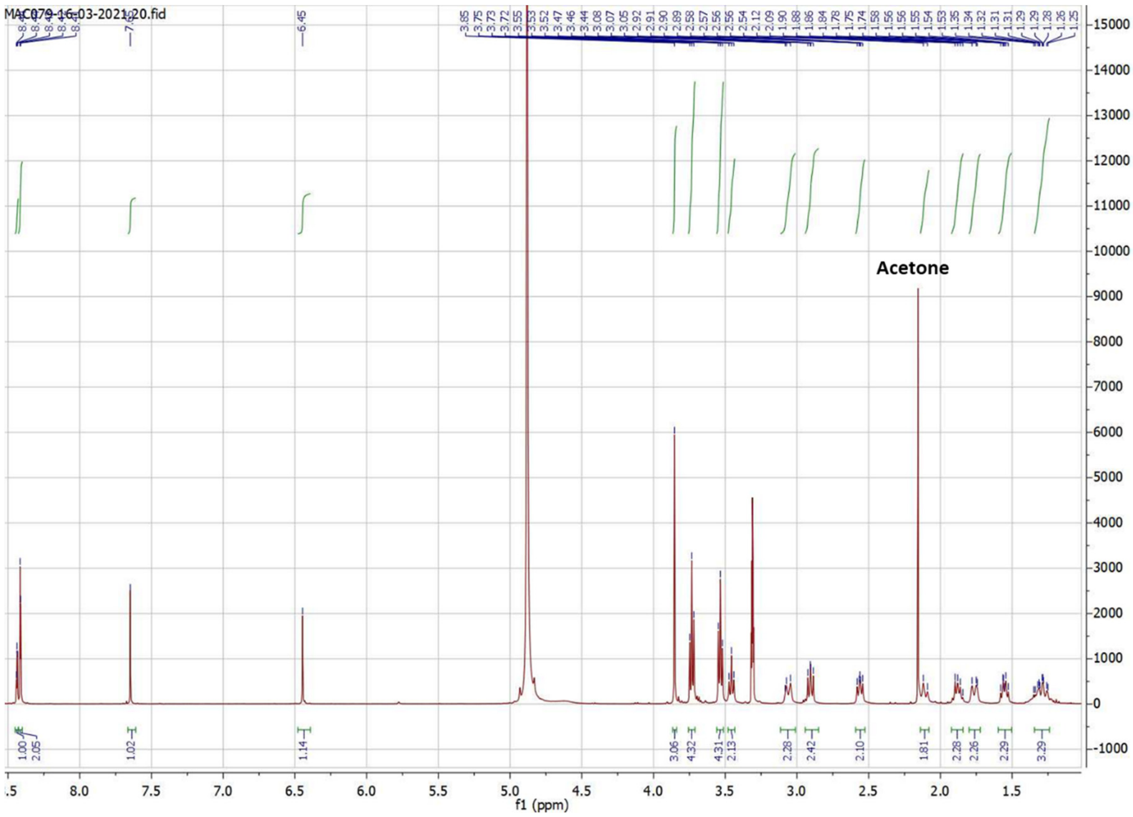
MAC079-16-03-2021.20.fid
Acetone
f1 (ppm)

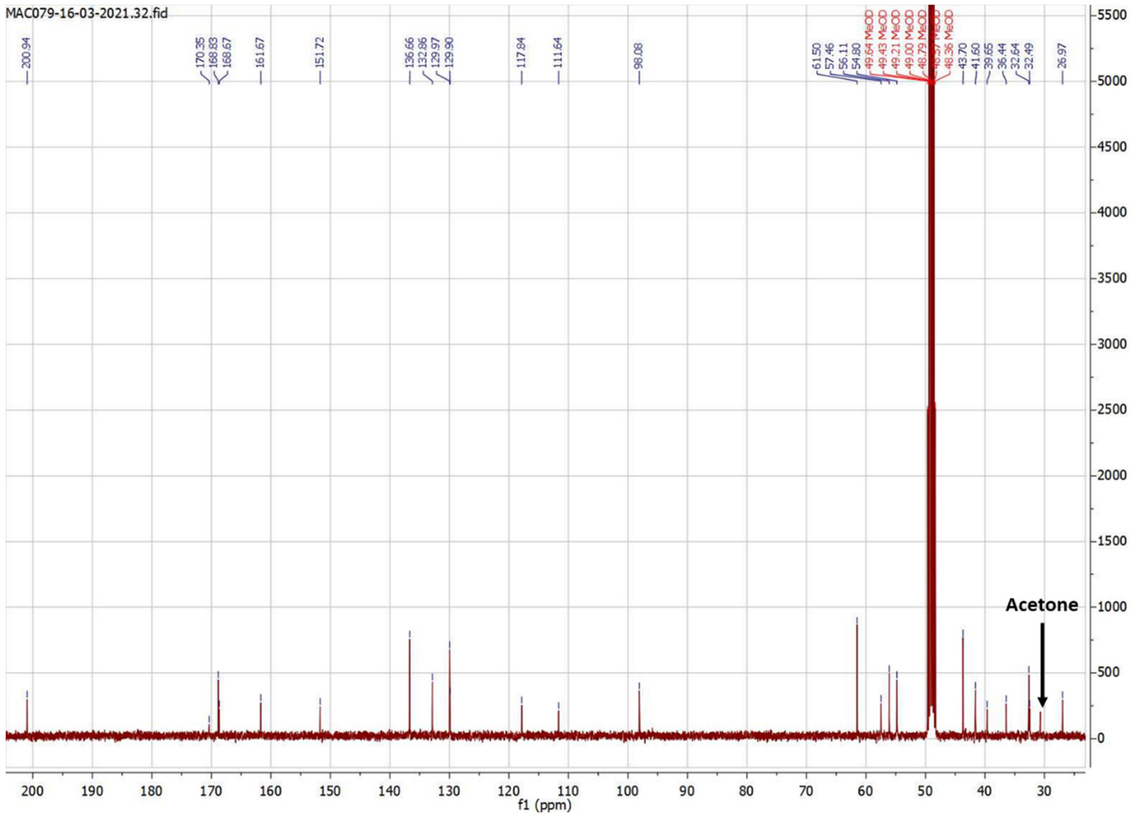
MAC079-16-03-2021.32.fid
Acetone
f1 (ppm)

Tert-butyl-(2-(2-(4-(3-(4-amino-5-chloro-2-methoxyphenyl)-3-oxopropyl)piperidin-1-yl)ethoxy)ethyl)carbamate (17)

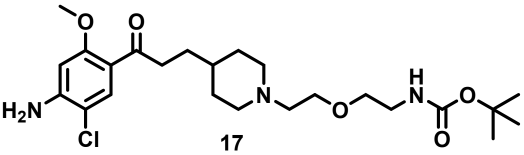

17

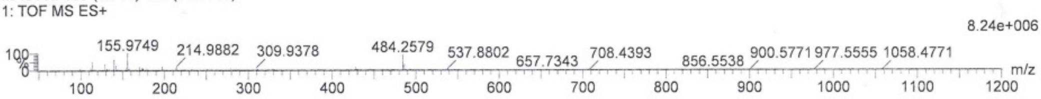

Elemental Composition Report **Page 1**

Single Mass Analysis
Tolerance = 5.0 PPM / DBE: min = -1.5, max = 50.0
Element prediction: Off
Number of isotope peaks used for i-FIT = 3

Monoisotopic Mass, Even Electron Ions
456 formula(e) evaluated with 1 results within limits (up to 50 closest results for each mass)
Elements Used:
C: 0-24 H: 0-100 N: 0-3 O: 0-5 Na: 0-1 Cl: 0-1 I: 0-1

MAC082 183 (1.715) Cm (183:188)
1: TOF MS ES+

Minimum:				-1.5				
Maximum:		5.0	5.0	50.0				
Mass	Calc. Mass	mDa	PPM	DBE	i-FIT	Norm	Conf(%)	Formula
484.2579	484.2578	0.1	0.2	6.5	1974.3	n/a	n/a	C24 H39 N3 O5 Cl

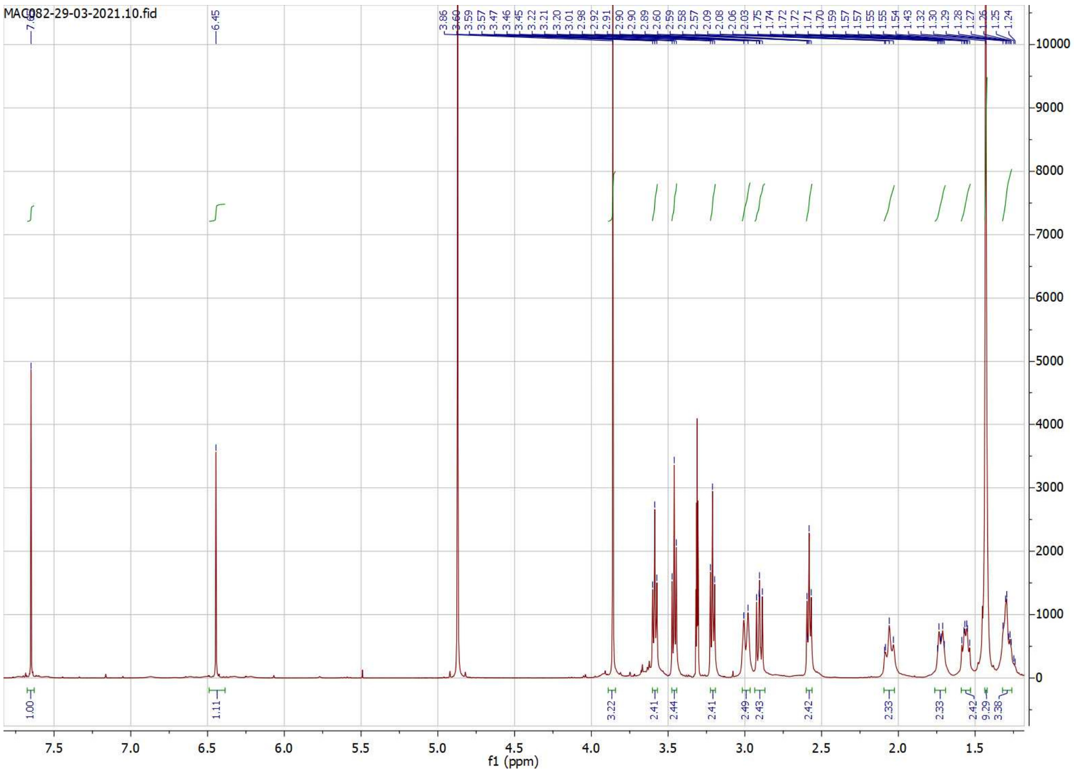

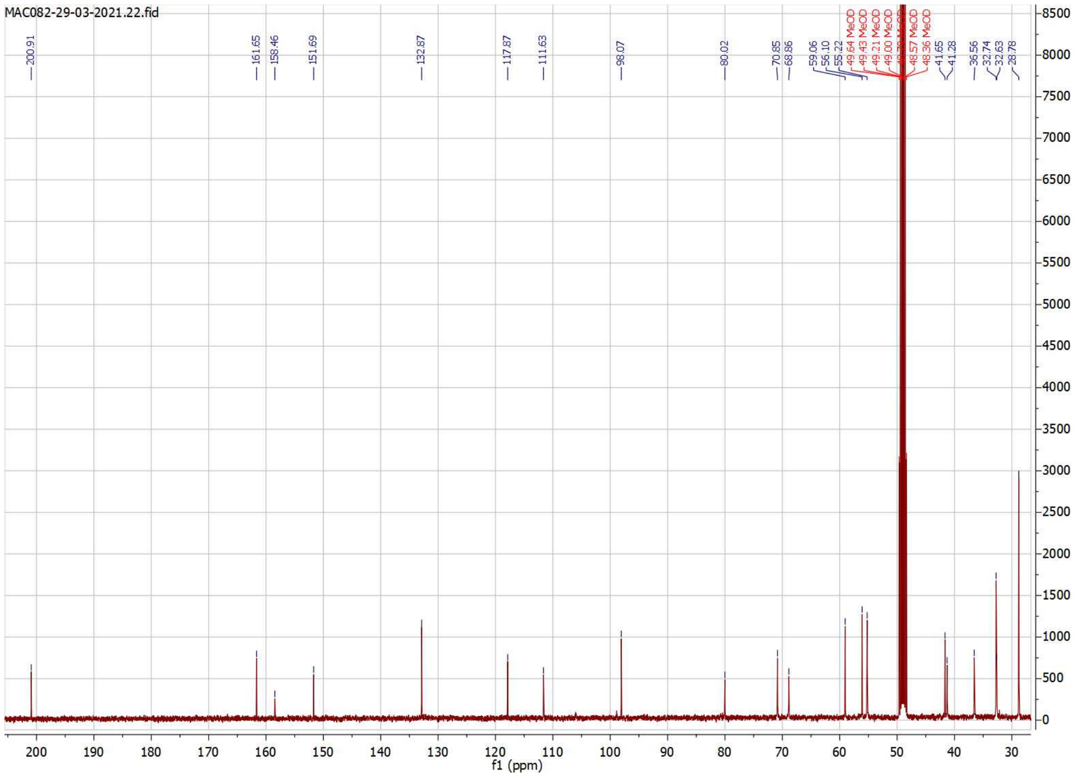

1,3-dimethyl-5-[(2(2-{4-[3-(4-amino-5-chloro-2-methoxyphenyl)-3-oxopropyl]piperidin-1-yl}ethoxy)ethyl)carbamoyl]benzene-1,3-dicarboxylate (18)

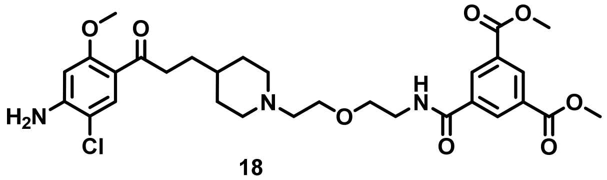

18

Elemental Composition Report **Page 1**

Single Mass Analysis
Tolerance = 5.0 PPM / DBE: min = -1.5, max = 50.0
Element prediction: Off
Number of isotope peaks used for i-FIT = 3

Monoisotopic Mass, Even Electron Ions
244 formula(e) evaluated with 1 results within limits (up to 50 closest results for each mass)
Elements Used:
C: 30-30 H: 0-40 N: 0-3 O: 0-8 Na: 0-1 Cl: 0-1 I: 0-1

MAC083 189 (1.768) Cm (189:193)
1: TOF MS ES+

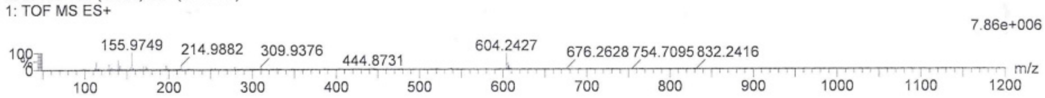

Minimum:				-1.5				
Maximum:		5.0	5.0	50.0				
Mass	Calc. Mass	mDa	PPM	DBE	i-FIT	Norm	Conf(%)	Formula
604.2427	604.2426	0.1	0.2	12.5	1739.7	n/a	n/a	C30 H39 N3 O8 Cl

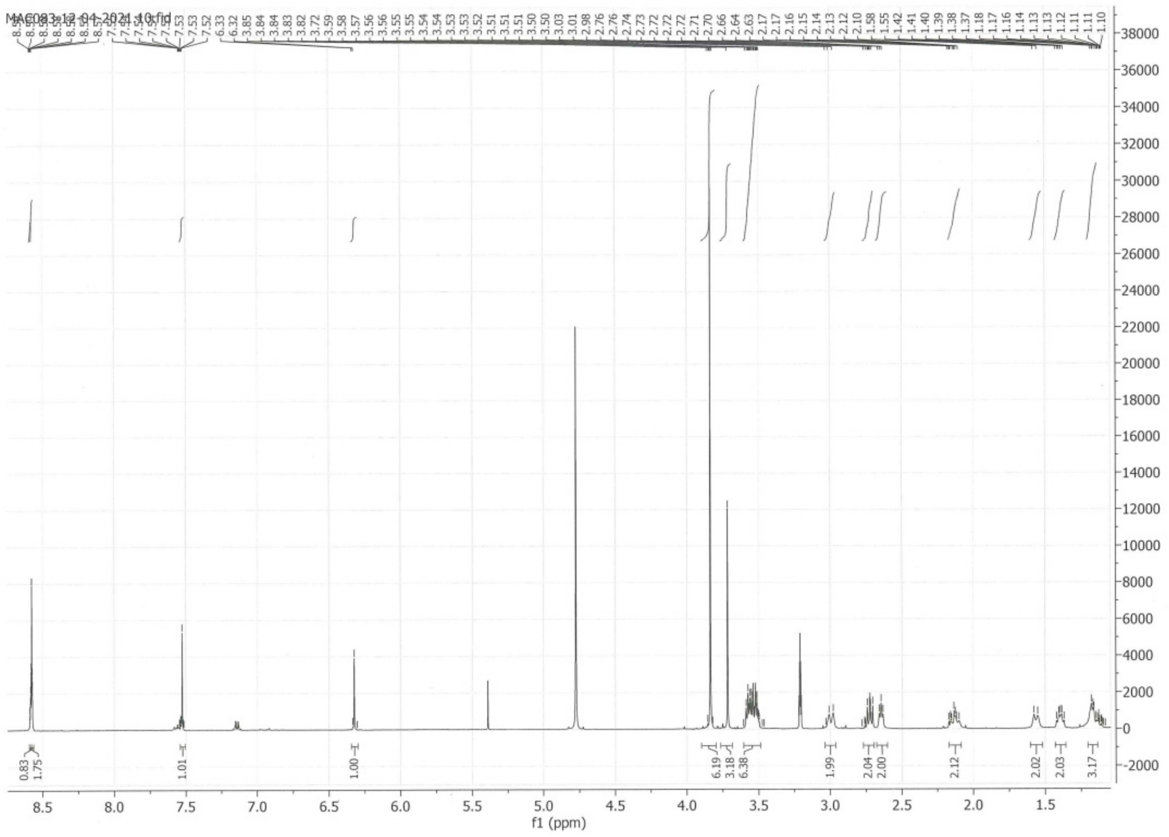
MAC083-12-04-2021.10.fid
f1 (ppm)

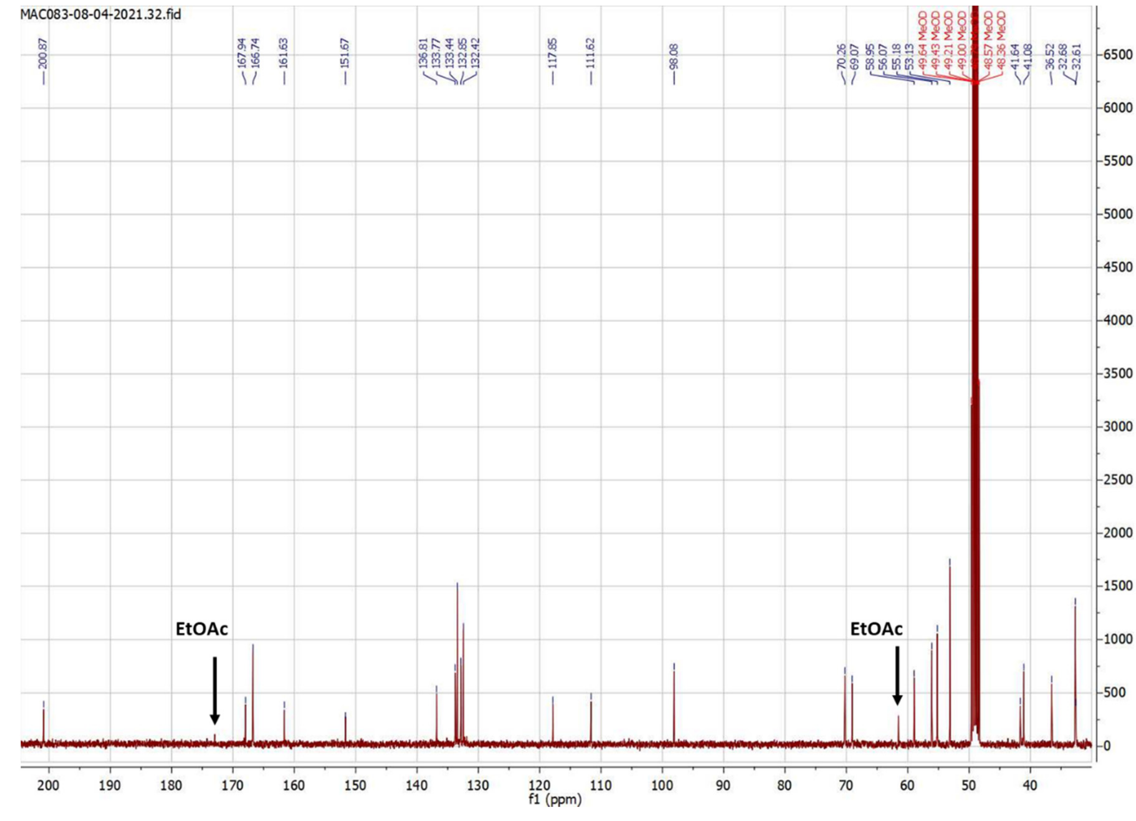
MAC083-08-04-2021.32.fid
EtOAc
EtOAc
f1 (ppm)

N[1]*-(2-(2-(4-(3-(4-amino-5-chloro-2-methoxyphenyl)-3-oxopropyl)piperidin-1-yl)ethoxy)ethyl)-N*[3]*,N*[5]*-bis(2-hydroxyethyl)benzene-1,3,5-tricarboxamide (19)*

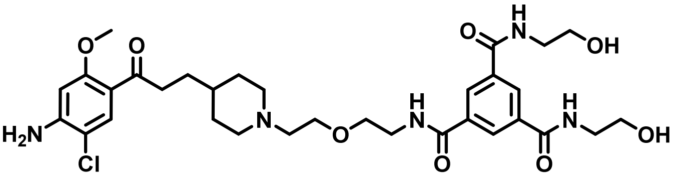

19 (ENT-C236)

Elemental Composition Report **Page 1**

Single Mass Analysis
Tolerance = 5.0 PPM / DBE: min = -1.5, max = 50.0
Element prediction: Off
Number of isotope peaks used for i-FIT = 3

Monoisotopic Mass, Even Electron Ions
473 formula(e) evaluated with 1 results within limits (up to 50 closest results for each mass)
Elements Used:
C: 30-32 H: 0-45 N: 0-5 O: 0-8 Na: 0-1 Cl: 0-1 I: 0-1

MAC084 155 (1.452) Cm (155:161)
1: TOF MS ES+

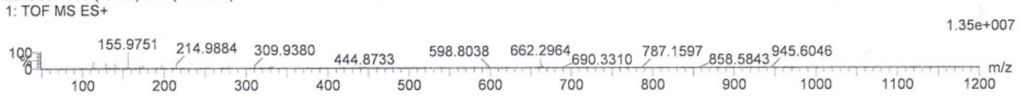

Mass	Calc. Mass	mDa	PPM	DBE	i-FIT	Norm	Conf(%)	Formula
Minimum:				-1.5				
Maximum:		5.0	5.0	50.0				
662.2964	662.2957	0.7	1.1	12.5	1891.7	n/a	n/a	C32 H45 N5 O8 Cl

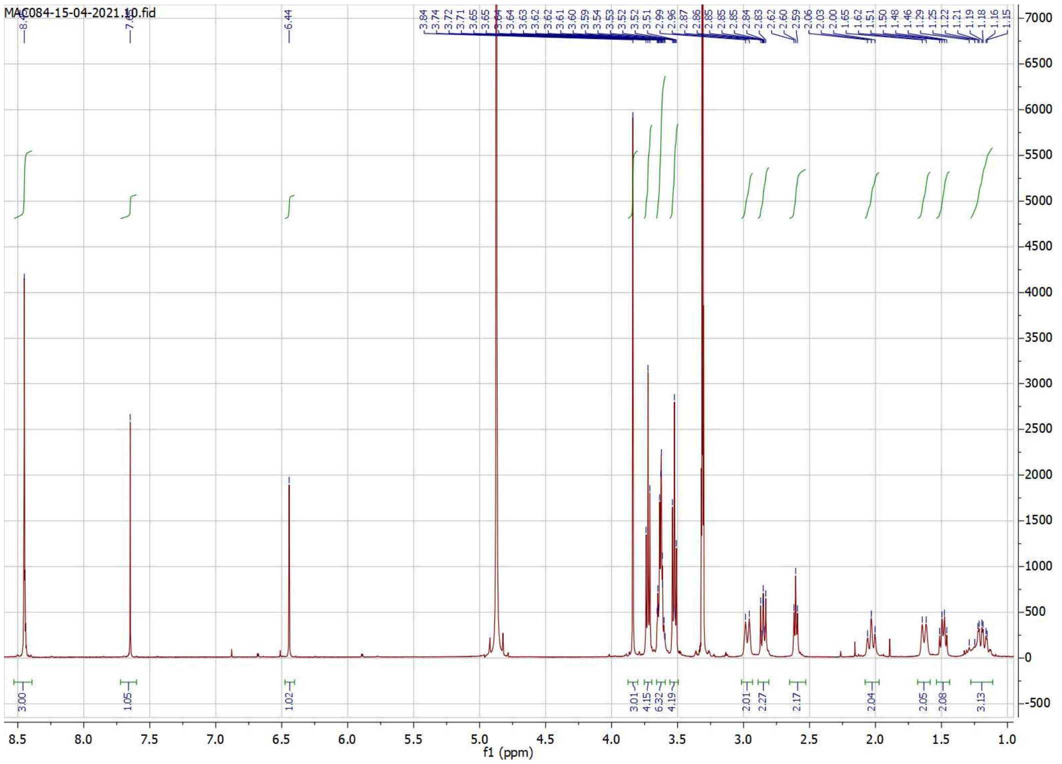
MAC084-15-04-2021.10.fid
f1 (ppm)

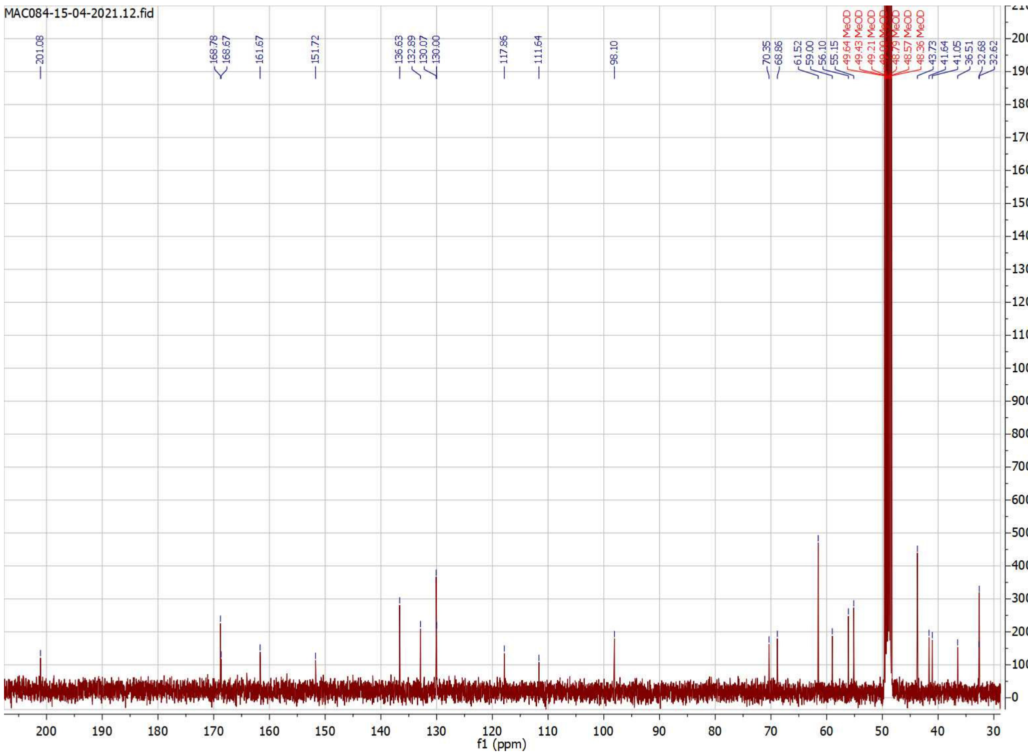
MAC084-15-04-2021.12.fid
f1 (ppm)

Full images of Western blot of phosphorylation assay of NIH-3T3 TrkB cell line

In parallel with described compounds other derivatives (named compound) have been tested along the main one in the experiment.

In the main text, on the Figure 4., it is shown the part of the Western blot from the Image 2.

Image 1.

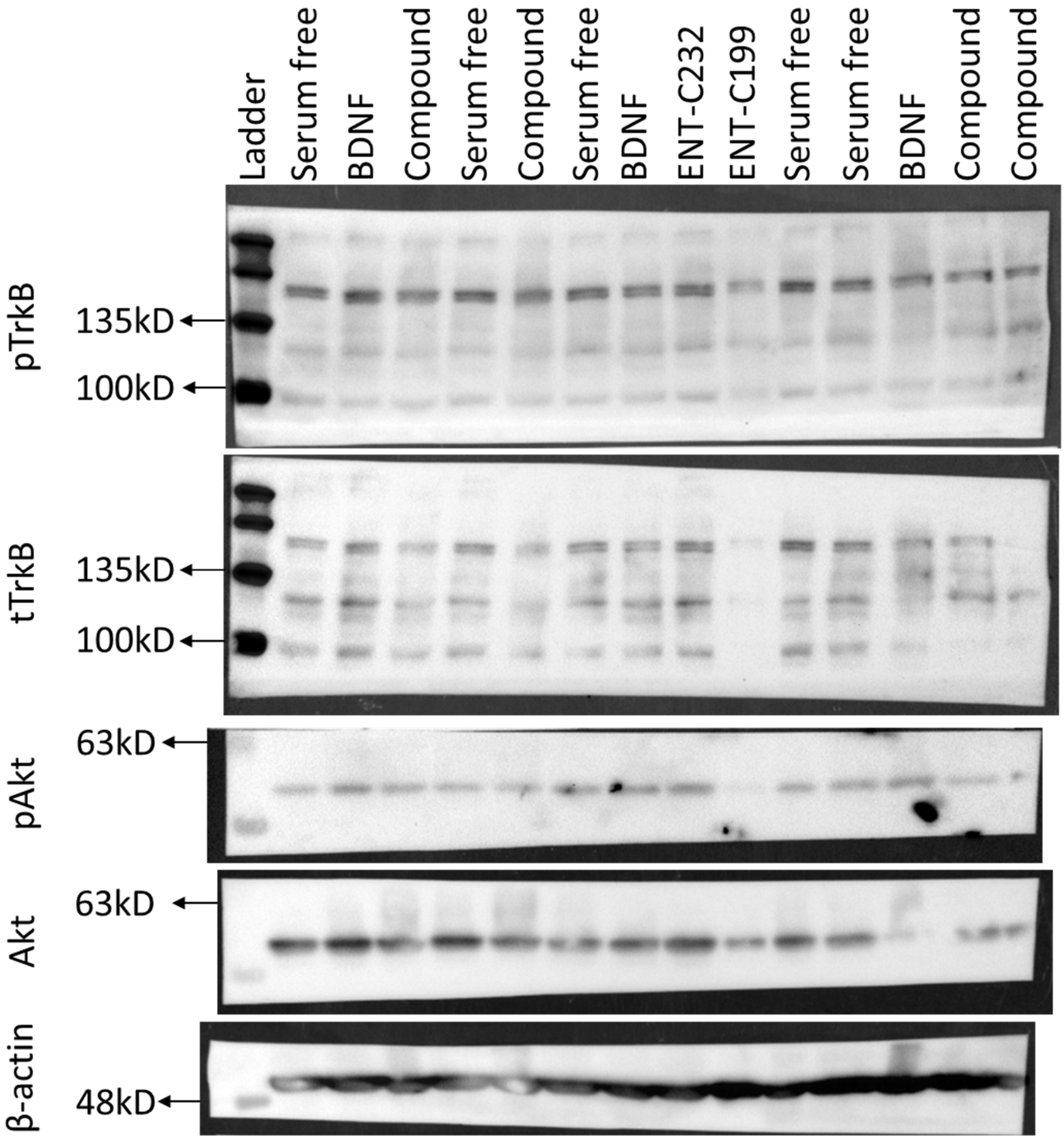

Image 2.

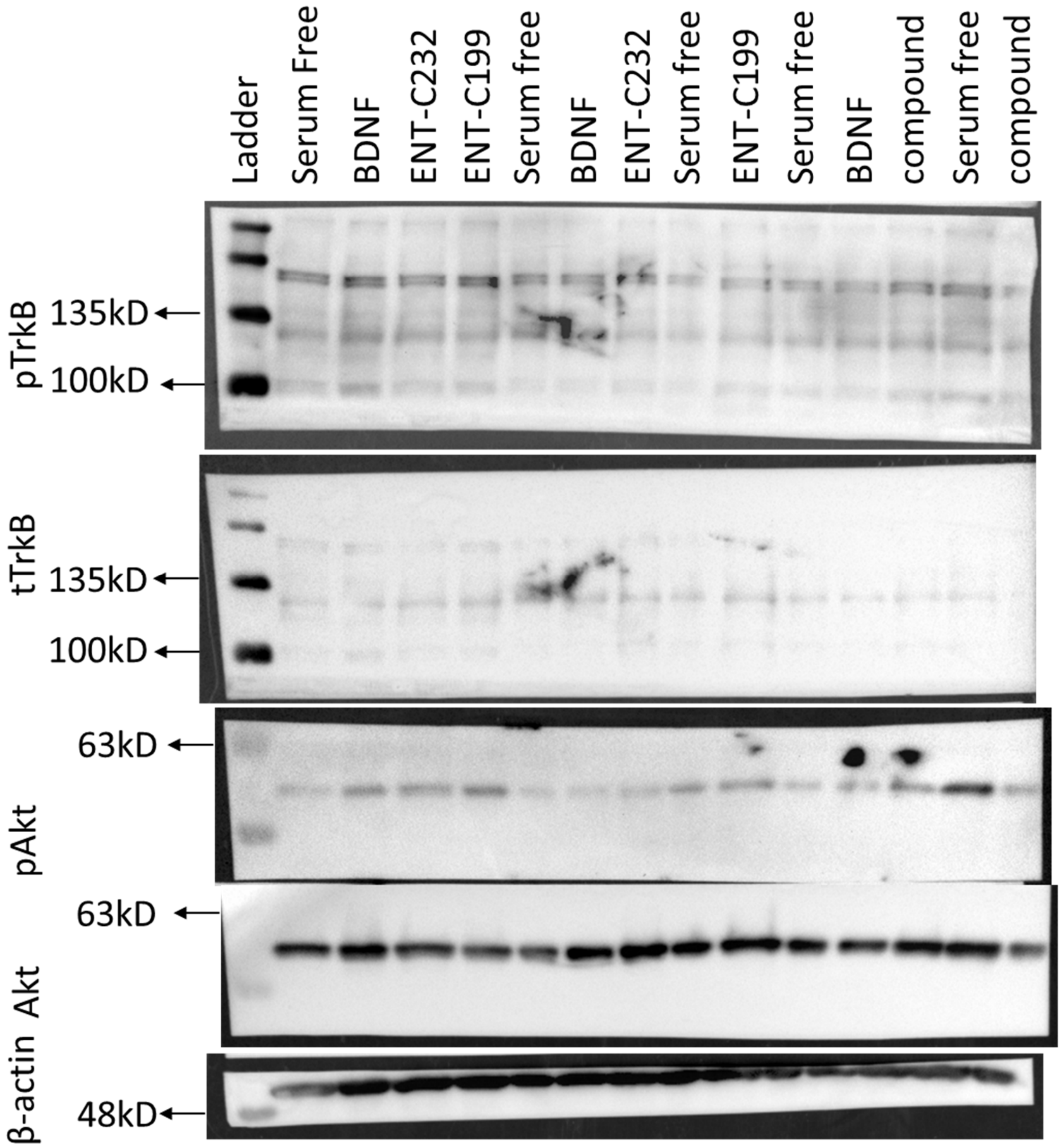

Ladder
Serum Free
BDNF
ENT-C232
ENT-C199
Serum free
BDNF
ENT-C232
Serum free
ENT-C199
Serum free
BDNF
compound
Serum free
compound
pTrkB
135kD
100kD
tTrkB
135kD
100kD
63kD
pAkt
63kD
Akt
β-actin
48kD

Image 3.

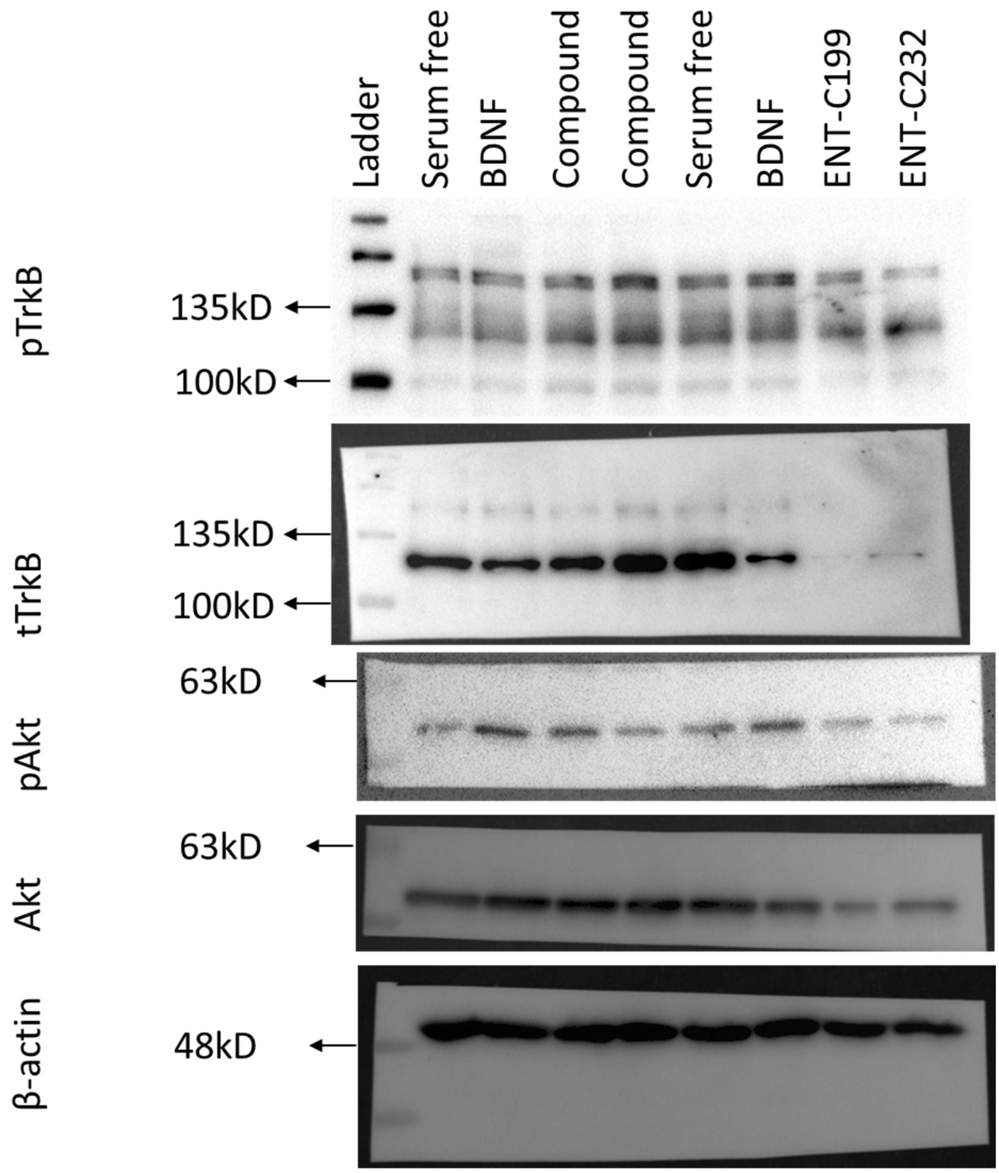
Ladder
Serum free
BDNF
Compound
Compound
Serum free
BDNF
ENT-C199
ENT-C232
pTrkB
135kD
100kD
tTrkB
135kD
100kD
pAkt
63kD
Akt
63kD
β-actin
48kD

Image 4.

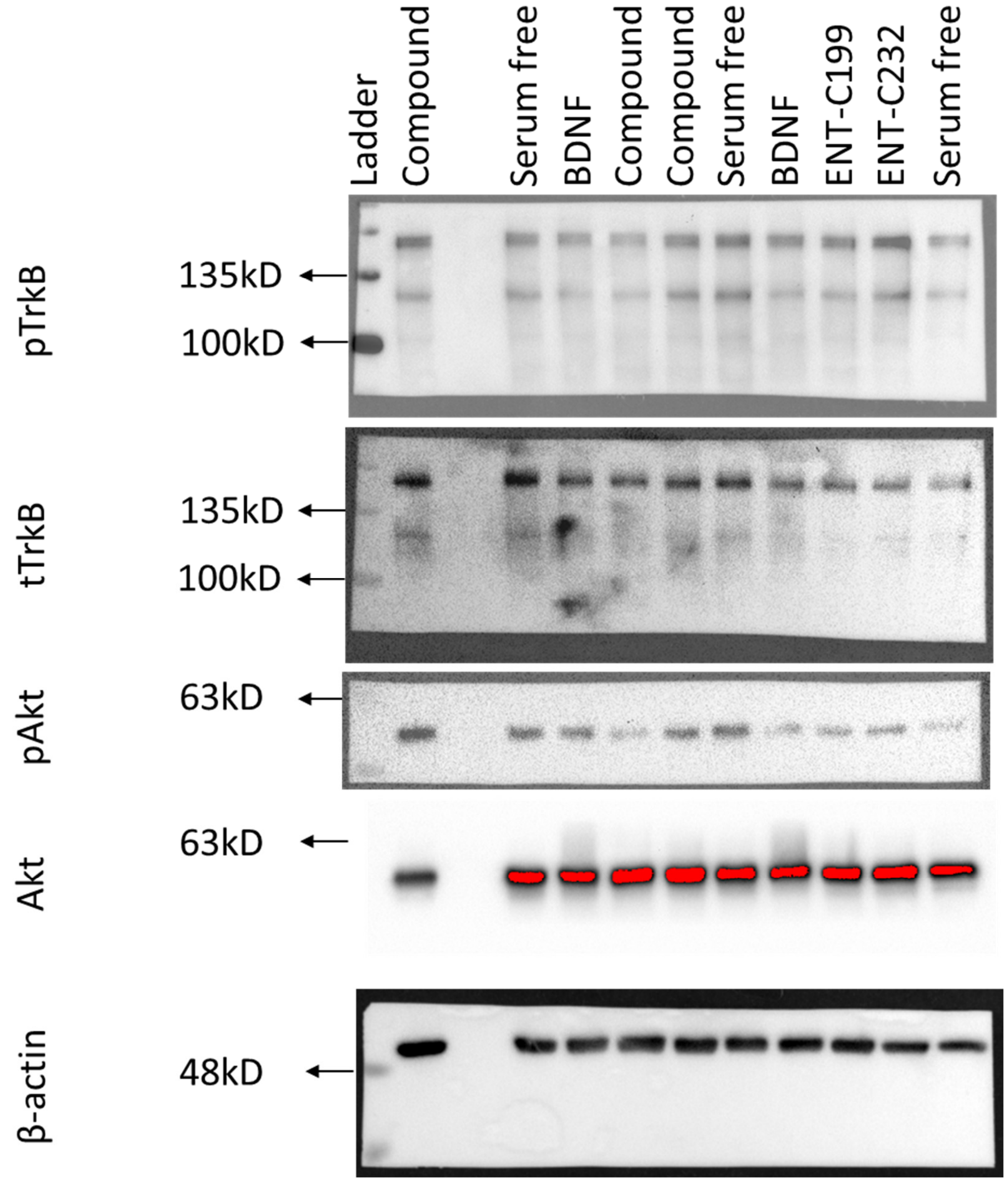
Ladder
Compound
Serum free
BDNF
Compound
Compound
Serum free
BDNF
ENT-C199
ENT-C232
Serum free
pTrkB
135kD
100kD
tTrkB
135kD
100kD
pAkt
63kD
Akt
63kD
β-actin
48kD

Determination of cAMP production

5-HT

	EC50	Log(EC50)	Top AMPc
TC27	2.20E-09	-8.7	100.00%
TC14	4.33E-09	-8.4	100.00%
TC15	3.70E-09	-8.4	100.00%
Moy.	**3.41E-09**	**-8.5**	**100%**
SEM	6.33E-10	0.1	0.00%

RS 67333

	EC50	Log(EC50)	Top AMPc
TC27	7.86E-10	-9.1	67.01%
TC14	1.54E-09	-8.8	60.01%
TC15	2.93E-10	-9.5	76.26%
Moy.	**8.72E-10**	**-9.1**	**68%**
SEM	3.62E-10	0.2	4.71%

ENT-C199

	EC50	Log(EC50)	Top AMPc
TC27	1.25E-09	-8.9	53.83%
TC14	3.96E-10	-9.4	46.75%
TC15	8.71E-10	-9.1	42.14%
Moy.	**8.40E-10**	**-9.1**	**48%**
SEM	2.48E-10	0.1	3.40%

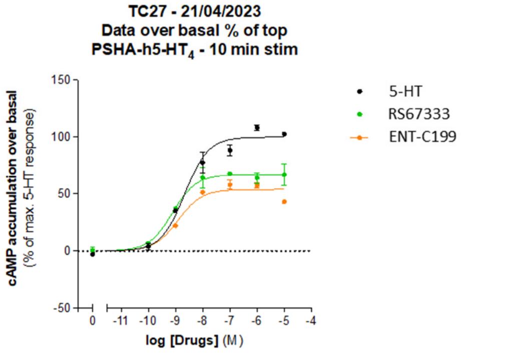

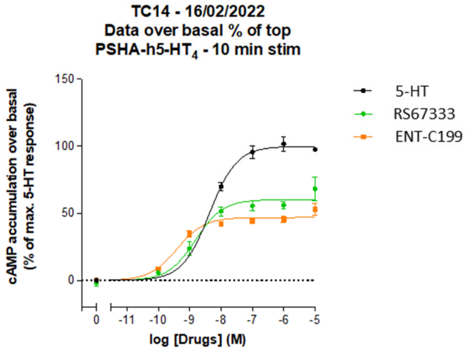

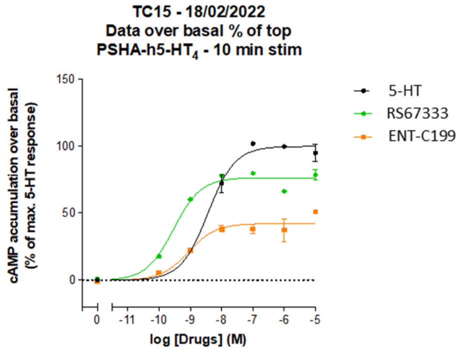

Supplement: Supplementary file 1 [file molecules-29-00515-s001.zip › molecules-2693547-supplementary.pdf]
